# Fluid resuscitation practices in cardiac surgery patients in the USA: a survey of health care providers

**DOI:** 10.1186/s13741-017-0071-6

**Published:** 2017-10-19

**Authors:** Solomon Aronson, Paul Nisbet, Martin Bunke

**Affiliations:** 1 Department of Anesthesiology, Duke University, 201 Trent Drive, 101 Baker House, Durham, NC 27710 USA; 2 One Research, LLC, 1150 Hungryneck Blvd. Suite C-303, Mt. Pleasant, SC 29464 USA; 3 Department of Medical Affairs, Grifols, 79 T.W. Alexander Drive, 4101 Research Commons, Research Triangle Park, Raleigh, NC 27709 USA

**Keywords:** Fluid resuscitation, Colloids, Crystalloids, Albumin, Cardiovascular surgery, Intraoperative volume expansion, Cardiopulmonary bypass, Survey

## Abstract

**Background:**

Fluid resuscitation during cardiac surgery is common with significant variability in clinical practice. Our goal was to investigate current practice patterns of fluid volume expansion in patients undergoing cardiac surgeries in the USA.

**Methods:**

We conducted a cross-sectional online survey of 124 cardiothoracic surgeons, cardiovascular anesthesiologists, and perfusionists. Survey questions were designed to assess clinical decision-making patterns of intravenous (IV) fluid utilization in cardiovascular surgery for five types of patients who need volume expansion: (1) patients undergoing cardiopulmonary bypass (CPB) without bleeding, (2) patients undergoing CPB with bleeding, (3) patients undergoing acute normovolemic hemodilution (ANH), (4) patients requiring extracorporeal membrane oxygenation (ECMO) or use of a ventricular assist device (VAD), and (5) patients undergoing either off-pump coronary artery bypass graft (OPCABG) surgery or transcatheter aortic valve replacement (TAVR). First-choice fluid used in fluid boluses for these five patient types was requested. Descriptive statistics were performed using Kruskal-Wallis test and follow-up tests, including *t* tests, to evaluate differences among respondent groups.

**Results:**

The most commonly preferred indicators of volume status were blood pressure, urine output, cardiac output, central venous pressure, and heart rate. The first choice of fluid for patients needing volume expansion during CPB without bleeding was crystalloids, whereas 5% albumin was the most preferred first choice of fluid for bleeding patients. For volume expansion during ECMO or VAD, the respondents were equally likely to prefer 5% albumin or crystalloids as a first choice of IV fluid, with 5% albumin being the most frequently used adjunct fluid to crystalloids. Surgeons, as a group, more often chose starches as an adjunct fluid to crystalloids for patients needing volume expansion during CPB without bleeding. Surgeons were also more likely to use 25% albumin as an adjunct fluid than were anesthesiologists. While most perfusionists reported using crystalloids to prime the CPB circuit, one third preferred a mixture of 25% albumin and crystalloids. Less interstitial edema and more sustained volume expansion were considered the most important colloid traits in volume expansion.

**Conclusions:**

Fluid utilization practice patterns in the USA varied depending on patient characteristics and clinical specialties of health care professionals.

**Electronic supplementary material:**

The online version of this article (10.1186/s13741-017-0071-6) contains supplementary material, which is available to authorized users.

## Background

Cardiac surgeries are commonly performed procedures that almost universally require fluid resuscitation during the intraoperative and perioperative period (Lange et al. 2011; Hirleman and Larson 2008; Verheij et al. 2006). The effects of fluid type, fluid amount, timing of fluid administration, and techniques for determining fluid responsiveness are actively debated topics (Lange et al. 2011; Cherpanath et al. 2014; van Haren and Zacharowski 2014). Specific disease and/or conditions of surgery involving cardiopulmonary bypass (CPB) and related patient pathophysiology have become increasingly recognized as key determinants for successful fluid resuscitation. For example, the balance between the hydrostatic pressure gradient which pushes water outward into the interstitial space and the colloid oncotic pressure (COP) which pulls water inward into the vessel (classic Starling’s Principle believed to govern fluid movement across the capillary wall) does not fully apply to conditions involving systemic inflammation and vascular barrier damage (Aditianingsih and George 2014; Jacob and Chappell 2013). Moreover, the endothelial glycocalyx layer (EGL), which comprises membrane-bound glycoproteins and proteoglycans with side chains of heparan sulfate, chondroitin, and dermatan sulfate, is recognized as an important factor in vascular barrier function (Jacob and Chappell 2013; Weinbaum et al. 2007; Becker et al. 2010; Myburgh and Mythen 2013). Whereas large molecules (e.g., albumin in colloid solutions) are retained inside the vessel, generating COP in the intravascular compartment (Aditianingsih and George 2014; Jacob and Chappell 2013; Myburgh and Mythen 2013); small molecules (e.g., electrolytes in crystalloid solutions) can travel freely through the vessel wall and thus can draw water into the interstitial space. Cardiopulmonary bypass can produce changes in fluid physiology and fluid responsiveness in patients (Lange et al. 2011; Hirleman and Larson 2008; Verheij et al. 2006), characterized by increased interstitial fluid as a consequence of decreased COP, damaged EGL, and inflammatory changes (Lange et al. 2011; Hirleman and Larson 2008; Jacob and Chappell 2013; Hoeft et al. 1991; Ortega-Loubon et al. 2015). This shift of fluid from the intravascular space to the interstitial space, in addition to blood and fluid losses during the surgical procedure, can result in an intravascular hypovolemia that requires fluid resuscitation.

A survey of current fluid usage by health care professionals (HCPs) involved in cardiovascular surgeries in the USA was developed to (a) examine the use of different fluid types for resuscitation (i.e., crystalloids, plasma-derived colloid [albumin], synthetic colloids [hydroxyethyl starches, HES]) in patients undergoing cardiovascular surgery, (b) determine whether certain patient characteristics and/or practice settings influence the type of fluid utilized for resuscitation, (c) determine whether the fluid selected for resuscitation varies by clinical specialties of the treating HCPs, and (d) determine the fluids used to prime the CPB circuit in patients undergoing on-pump procedures.

## Methods

### Study design

This study was cross-sectional and collected survey data from 124 cardiothoracic surgeons, cardiovascular anesthesiologists, and perfusionists to investigate the patterns of fluid utilization in cardiovascular surgery. The online survey was conducted November 4 through 17, 2015, with an average survey completion time of 9 min. The 38-item self-administered questionnaire (Additional file 1) obtained information on fluids used for hemodynamic management in the operating room and in the first 24-h postoperative period, as well as on volume status indicators most often used to determine volume expansion needs. Survey participants were presented with five different hypothetical patient scenarios encountered frequently in cardiovascular surgery and asked to identify their first choice of fluid for volume expansion for each patient type from a list of colloid and crystalloid fluids. The five patient scenarios were:

1. Volume expansion during CPB when not experiencing significant blood loss
2. Volume expansion in the presence of blood loss during CPB when blood transfusion is not indicated (adequate hemoglobin [Hb])
3. Volume maintenance during acute normovolemic hemodilution (ANH, autologous blood collection)
4. Volume expansion while patients were supported with extracorporeal membrane oxygenation (ECMO) or a ventricular assist device (VAD)
5. Intraoperative volume expansion for off-pump coronary artery bypass graft (OPCABG) surgery or transcatheter aortic valve replacement (TAVR)

The six types of fluids were crystalloids, 5% albumin, 25% albumin, first-generation HES or HES 450/600 (e.g., 6% HES 450/0.70 and 6% HES 600/0.75), third-generation HES or HES 130 (e.g., 6% HES 130/0.4), and blood-derived blood products other than albumin. The participants rated the frequency with which they used various fluid types for volume expansion using a 5-point scale (from 1 for “always” to 5 for “never”). Participants were also asked to indicate the bolus volumes (mL) of the crystalloids and of colloids that they typically use for volume expansion. Participants rated the importance of certain colloid characteristics (e.g., more sustained volume expansion, faster volume expansion) and non-oncotic properties of albumin (e.g., transport of metabolites, free radical scavenging) on the patient treatment decision-making process using a 5-point scale (from 1 for “not important” to 5 for “absolutely essential”). Four of the 38 questions addressed pump priming preferences for CPB circuits and were asked of perfusionists only.

### Participants

A total of 124 participants were recruited from the e-Rewards Medical panel. e-Rewards Medical is a leading provider of market research services to the professional health care community. The panel consists of HCPs who have opted to become members of the panel and were paid for their time. Email invitations for participation in this study were sent from e-Rewards to the non-probability sample of its panelists, meaning this sample set was not a random selection of all physicians. Of note, the panelists remained anonymous to the investigators in this study. The email invitation provided a general description of the survey topic (i.e., “Fluid and Hemodynamic Management”) and a link by which to access the online survey. Each invitation contained a unique identifier that prevented any one respondent from taking the survey more than once. To qualify for participation, respondents had to specialize in cardiac surgery, adult cardiovascular anesthesiology, or be a perfusionist; had been in practice for at least 2 years since residency or training in the USA but not in the states of Minnesota, Vermont, West Virginia, Massachusetts, nor the District of Columbia as these states prohibit or limit compensation to physicians; and had performed or were involved in at least four cardiac bypass surgeries per month. Anesthesiologists who specialized in pediatrics were excluded because fluid management for pediatrics is different than for adults due to vast differences in the pathophysiology of their circulatory system. To ensure a minimum number of completed surveys were received from each group, subquotas were set for each clinical specialty: 50 surgeons, 50 anesthesiologists, and 50 perfusionists.

This research project involved obtaining the opinions of physicians and perfusionists about their choices for the use of various fluids for volume expansion in five different hypothetical patient situations. No patient data were obtained, and no questions were asked of the participants that would help in identifying them. All participant data were de-identified. Hence, this study was exempt from requiring institutional review board approval under United States Code of Federal Regulations Title 45 Part 46.101(b)(2) by Copernicus Group Independent Review Board (CGIRB). The study did receive a formal Letter of Exemption from the CGIRB.

### Statistical analyses

Most questions were based on 5-point scales and provided ordinal data which, by definition, are not normally distributed. As such, descriptive statistics were performed using a Kruskal-Wallis test to evaluate differences among the respondent groups on the ordinal measures. Follow-up tests were conducted to evaluate differences within and between patient scenarios. *T* tests were used to evaluate differences across ratio variables (i.e., bolus volume). Statistical significance was assessed at the alpha level of less than 0.05. Descriptive analyses were performed using SPSS (Version 23.0).

## Results

### Participant characteristics

Of the 124 HCPs who completed the survey, 52 (41.9%) were anesthesiologists, 47 (37.9%) were surgeons, and 25 (20.2%) were perfusionists (Table 1). The primary practice setting for most HCPs was a non-university hospital (73.4%) followed by university hospital (26.6%). The average number of bypass surgeries that the HCPs participated in per month was 28.6 for surgeons, 24.7 for anesthesiologists, and 21.4 for perfusionists.

**Table 1:** Characteristics of survey respondents (*n* = 124)

	Anesthesiologists^A^	Surgeons^S^	Perfusionists^a^
	(*n* = 52)	(*n* = 47)	(*n* = 25)
No. of cardiac bypass per month, mean (STD)	24.7 (21.1)	28.6 (20.2)	21.0 (13.5)
Median	17.5	25.0	20.0
(Range)	(4–100)	(8–100)	(7–60)
Primary practice setting			
Non-university hospital, %	78.8%	68.1%	72.0%
University hospital, %	21.2%	31.9%	28.0%
No. of beds in primary hospital, mean (STD)	476 (234.0)	486 (215.3)	513 (362.1)
Median	400	450	425
(Range)	(99–1500)	(150–1000)	(200–2000)
Years since residency/training, mean (STD)	14.3 (7.5)	20.6^A^ (10.7)	22.1 (7.8)
Median	13.5	20.0	24.0
(Range)	(3–37)	(3–50)	(10–37)

Superscripts A and S denote differences between anesthesiologists and surgeons that are statistically significant at *P* < .05

^a^Statistical tests were not performed on data for perfusionists due to small sample size

### Survey data

**Fig. 1:**
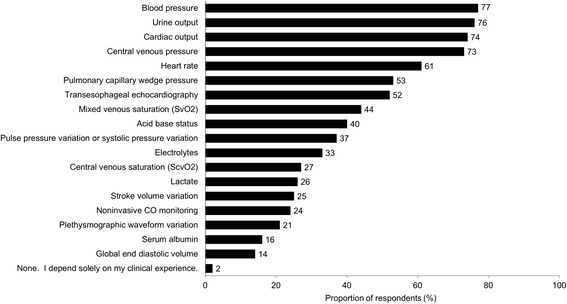
Use of volume status indicators/diagnostic tools in assessing fluid needs^a^ (*n* = 124^b^). ^a^Responses to the following question: Which of the following indicators (diagnostic tools) of volume status and the need for volume expansion do you use? (Please select all that apply.) ^b^Sample included 52 anesthesiologists, 47 surgeons, and 25 perfusionists

The five most commonly used indicators of volume status were blood pressure (77%), urine output (76%), cardiac output (74%), central venous pressure (73%), and heart rate (61%) (Fig. 1). Pulmonary capillary wedge pressure was used by 53% of HCPs as indicators of volume status, and transesophageal echocardiography was used by 52%. A statistically significant difference for volume indicator use was found between surgeons and anesthesiologists for transesophageal echocardiography (26 vs 79%, respectively, *P* < .001), pulse pressure variation (26 vs 56%, respectively, *P* = .002), and stroke volume variation (15 vs 44%, respectively, *P* = .001).

**Fig. 2:**
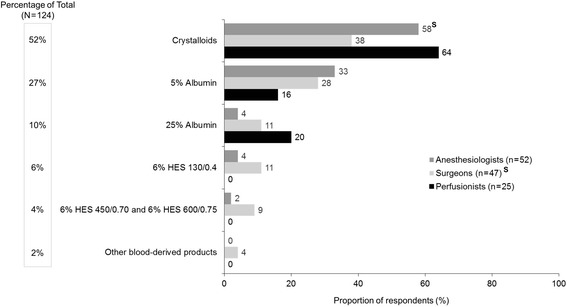
First choice of intravenous fluid for patients needing volume expansion during CPB when not experiencing significant blood loss^a^ (scenario 1, *n* = 124). CPB, cardiopulmonary bypass; HES, hydroxyethyl starch. Superscript S denotes the difference between specialties that are statistically significant at *P*  <  .05. ^a^Responses to the following question: Which of the following is your first choice for a patient who needs volume expansion during cardiovascular surgery with CPB who is not experiencing significant blood loss?

The first choice of intravenous (IV) fluid for a patient needing volume expansion during CPB when not experiencing significant blood loss (scenario 1) was crystalloids, followed by 5% albumin and 25% albumin, respectively (Fig. 2). In this patient scenario, crystalloids were used more frequently as the fluid of first choice by anesthesiologists (58%) compared to surgeons (38%, *P* = 0.054). Higher percentages of surgeons than anesthesiologists chose HES and blood-derived products other than albumin, while no perfusionists chose any of those fluid types. The most frequently used adjunct fluid to crystalloids was 5% albumin (Additional file 2: Figure S1).

**Fig. 3:**
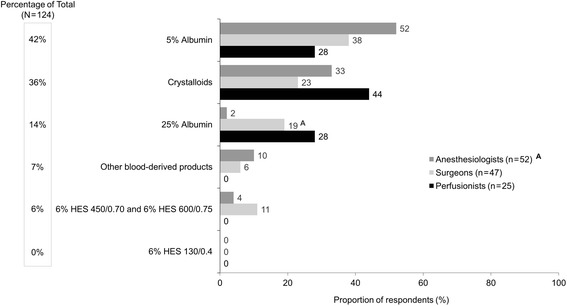
First choice of IV fluid for patients needing volume expansion in the presence of blood loss during CPB when blood transfusion is not indicated (adequate Hb)^a^ (scenario 2, *n* = 124). CPB, cardiopulmonary bypass; Hb, hemoglobin; HES, hydroxyethyl starch; IV, intravenous. Superscript A denotes the difference between specialties that are statistically significant at *P* < .05. ^a^Responses to the following question: Which of the following is your first choice for a patient who needs volume expansion in the presence of blood loss when blood transfusion is not indicated (adequate Hb) during cardiovascular surgery with CPB?

For patients needing volume expansion in the presence of blood loss during CPB when blood transfusion is not indicated (adequate Hb, scenario 2), HCPs chose 5% albumin most frequently as the first choice of IV fluid (Fig. 3). Crystalloid was the second most frequently chosen fluid, followed by 25% albumin. Surgeons chose 25% albumin significantly more often than anesthesiologists (19 vs 2%, respectively, *P* < .05), while no HCP chose HES 130. When the first fluid choice was 5% albumin, the most frequently chosen adjunct fluid was crystalloids (Additional file 3: Figure S2).

**Fig. 4:**
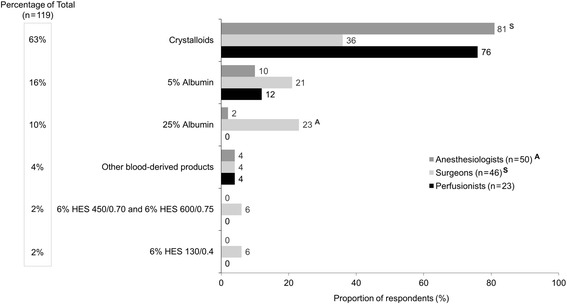
First choice of IV fluid for maintenance during ANH (autologous blood collection)^a^ (scenario 3, *n* = 119^b^). *ANH*, acute normovolemic hemodilution; HES, hydroxyethyl starch; IV, intravenous; n/a, not available. Superscript A and S denote differences between specialties that are statistically significant at *P* <  .05. ^a^Responses to the following question: Which of the following is your first choice for a patient for volume replacement maintenance during ANH (autologous blood collection)? ^b^A total of 119 HCPs responded to this question; five respondents (two anesthesiologists, one surgeon, two perfusionists) indicated the patient type was “not applicable” to their practice

Similar to scenario 1, the first choice of IV fluid for volume maintenance during ANH (scenario 3) was crystalloids, followed by 5% albumin and then 25% albumin (Fig. 4). Anesthesiologists chose crystalloids significantly more often than surgeons did (81 vs 36%, respectively, *P* < .05) for volume maintenance during ANH. Again, 5% albumin was the most frequently used adjunct fluid to crystalloids (Additional file 4: Figure S3).

**Fig. 5:**
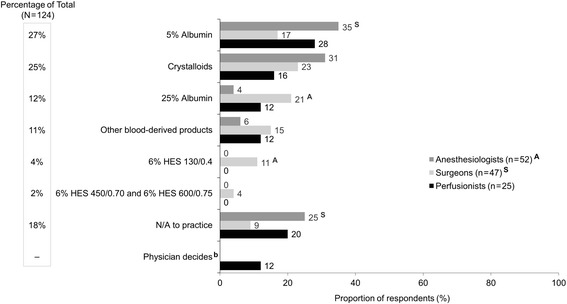
First choice of IV fluid for volume expansion during ECMO or VAD^a^ (scenario 4, *n* = 124). ECMO, extracorporeal membrane oxygenation; HES, hydroxyethyl starch; IV, intravenous; VAD, ventricular assist device. ^a^Responses to the following question: Which of the following is your first choice for a patient who needs volume expansion during ECMO or VAD? ^b^Response was only available to perfusionists

For volume expansion, while patients were supported by ECMO or a VAD (scenario 4), HCPs preferred 5% albumin and crystalloids equally as the first choice of IV fluid (Fig. 5). While anesthesiologists seemed to prefer 5% albumin more often than surgeons (35 vs 17%, respectively, *P* < .05), more surgeons preferred 25% albumin than anesthesiologists (21 vs 4%, respectively, *P* < .05). Only surgeons utilized HES fluids for this scenario. When the first fluid choice was 5% albumin, crystalloid was the most frequently chosen adjunct fluid (Additional file 5: Figure S4A), and 5% albumin was the most frequently chosen adjunct fluid when crystalloid was the first fluid choice (Additional file 5: Figure S4B). It is worth noting that 18% of the clinical practices in this survey did not utilize ECMO or VADs (Fig. 5).

**Fig. 6:**
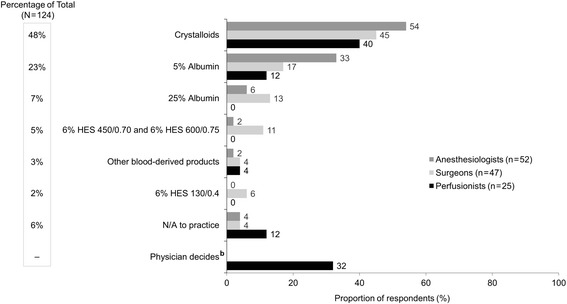
First choice of IV fluid for intraoperative volume expansion for OPCABG or TAVR^a^ (scenario 5, *n* = 124). HES, hydroxyethyl starch; IV, intravenous; OPCAB, off-pump coronary artery bypass surgery; TAVR, transcatheter aortic valve replacement. ^a^Responses to the following question: Which of the following is your first choice for a patient who needs intraoperative volume expansion for OPCAB or TAVR? ^b^Response was only available to perfusionists

As was seen in scenarios 1 and 3, a similar trend was observed for intraoperative volume expansion during OPCABG or TAVR (scenario 5). Crystalloid fluid was the most preferred first choice of IV fluid, followed by 5% albumin and then 25% albumin (Fig. 6), and 5% albumin was the adjunct fluid of choice when the first fluid choice was crystalloids (Additional file 6: Figure S5).

**Fig. 7:**
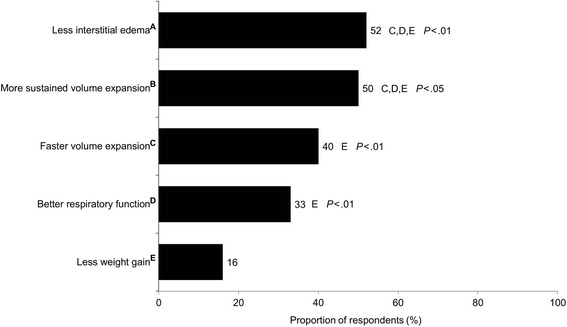
Importance of colloid traits when colloids were used for volume expansion^a^ (*n* = 124). Superscripts A–E on y-axis labels represent the respective trait for statistical comments. Letters following values represent the traits from which the trait’s percentage differs significantly. ^a^Responses to the following question: Using the scale below, please indicate how important each of the following is in terms of your reasons for using colloids for volume expansion (5-point scale: not important, somewhat important, important, very important, absolutely essential). Data presented in this graph are the proportions of respondents indicating colloid trait is “very important” or “absolutely essential” when used for volume expansion

**Fig. 8:**
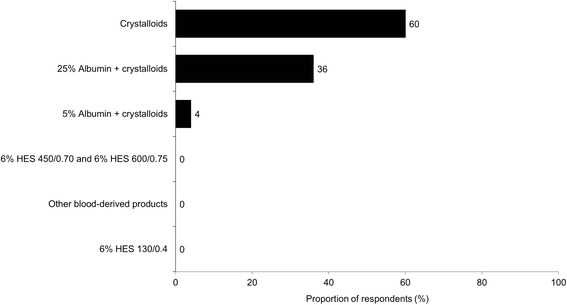
First choice of solutions for priming the CPB circuit among perfusionists^a^ (*n* = 25). CPB, cardiopulmonary bypass; HES, hydroxyethyl starch. ^a^Responses to the following question: Which of the following solutions is your first choice for priming the CPB circuit?

The most important colloid traits that influenced the decision to use colloids for volume expansion seemed to be “less interstitial edema” and “more sustained volume expansion” (Fig. 7). Most perfusionists (60%) preferred crystalloids as the priming solution for the CPB circuit, and approximately one third chose a mixture of 25% albumin and crystalloids (Fig. 8). One in five perfusionists have never used albumin to prime the CPB. The average volume of priming solution used by perfusionists was 1085 mL (median 1000 mL; range 500–2000 mL). In general, physicians reported the typical volume of colloid bolus as smaller (413–514 mL) than the volume of crystalloid bolus (620–670 mL). Physicians also reported having a higher level of influence (42–43%) on the decision to use albumin for volume expansion than perfusionists did (20%). The most common reasons given by physicians for not using 5% albumin were that it has a relatively higher cost relative to other fluids and that there is a lack of evidence for greater efficacy with albumin than with crystalloids. Perfusionists frequently mentioned that 5% albumin is often not available in their practices.

## Discussion

This cross-sectional study provided insights on the patterns of fluid utilization in cardiovascular surgery from a survey of 52 cardiovascular anesthesiologists, 47 cardiothoracic surgeons, and 25 perfusionists in the USA. This survey examined the fluids chosen for volume resuscitation by these 124 HCPs to treat five different hypothetical patient scenarios. The 25 perfusionists were surveyed to determine the solutions that they utilized to prime the CPB circuit. There were remarkable variability in clinical practice and a lack of consensus about the use of various fluid types in cardiovascular surgery patients.

In this survey, the most commonly preferred indicators of volume status were blood pressure, urine output, cardiac output, central venous pressure, and heart rate. Anesthesiologists preferred transesophageal echocardiography, pulse pressure variation, and stroke volume variation as indicators of volume status significantly more frequently than surgeons. Different fluid types were chosen as the first choice of IV fluids depending on the clinical context of the patients. For example, crystalloid fluid was the predominant first choice for patients needing volume expansion during CPB without bleeding (scenario 1), for fluid maintenance during ANH (scenario 3), and for intraoperative volume expansion during OPCABG or TAVR (scenario 5). On the other hand, 5% albumin was the primary fluid choice for patients needing volume expansion in the presence of blood loss during CPB not requiring transfusion (scenario 2) or during ECMO or VAD (scenario 4). When choosing colloids for volume expansion, HCPs felt that “less interstitial edema” and “more sustained volume expansion” were more important colloid traits than “faster volume expansion,” “better respiratory function,” and “less weight gain.” “Other blood-derived products” were chosen infrequently (less than 10%) as the first choice for fluid resuscitation by both university- and non-university-affiliated physicians; the only situation in which it was chosen by more than 10% of the respondents was for patients on ECMO and VAD.

In addition, there were differences in practice patterns among the clinical specialties of the treating HCPs. Significantly larger numbers of surgeons, relative to anesthesiologists, preferred 25% albumin for patients who need volume expansion in the presence of blood loss (scenario 2), for fluid maintenance during ANH (scenario 3), and for volume expansion during EMCO or VAD (scenario 4). Sixty percent of the perfusionists in this study preferred to use only crystalloids for priming the CPB circuit, while the other 40% used a mixture of albumin and crystalloids. The data from this study demonstrate that volume repletion practices vary dramatically and clinical data do not allow for consensus recommendations. While there are no large, randomized trials available to inform HCPs of definitive protocols for optimal volume resuscitation during cardiovascular surgeries, it is well accepted that fluid overload increases the risk of major complications after CABG (Morin et al. 2011). Whereas several studies (Hoeft et al. 1991; Russell et al. 2004; Sade et al. 1985; Kuitunen et al. 2004) have suggested that the use of colloid fluids in the priming solution during CPB is beneficial for maintaining COP.

Additional data indicate that utilization of colloid fluid results in a larger intravascular volume expansion than an equal volume of crystalloid fluid (Verheij et al. 2006; Finfer et al. 2011; Jacob et al. 2012; Skhirtladze et al. 2014), and the role of the EGL may be important. Studies have suggested that the EGL becomes damaged in numerous systemic inflammatory states (i.e., ischemia-reperfusion injury (Rehm et al. 2007), trauma (Johansson et al. 2011), and sepsis (Steppan et al. 2011; Ait-Oufella et al. 2010)), which may lead to interstitial edema (Aditianingsih and George 2014; Myburgh and Mythen 2013). In such conditions, colloids may behave more like crystalloids when there is significant damage to the EGL, and several large studies (Finfer et al. 2011; Myburgh et al. 2012; Annane et al. 2013; Perner et al. 2012) have failed to show any benefit from colloids in this context. On the other hand, two randomized trials in patients undergoing cardiac surgery support the suggestion that smaller volumes of colloids (HES and albumin) are required for volume resuscitation when compared to crystalloid solutions (Verheij et al. 2006; Skhirtladze et al. 2014). In this context, it has been argued that the addition of colloids to crystalloid mixture may theoretically have benefits over crystalloids alone (Roger et al. 2014). In a retrospective, hospital-discharge database study of 19,578 cardiac surgery patients, albumin administration for volume expansion during CABG appeared to be associated with a 25% reduction in postoperative mortality, relative to other non-protein colloids (Sedrakyan et al. 2003). However, a recent retrospective study of one cardiac surgery program comparing outcomes from 9 months before vs 3 months after its protocol changed to restrict the use of albumin in patients who required more than 3-L crystalloids within the first 24 h, had an albumin concentration < 3.0 g/dL, or had volume overload, found that there was no difference in morbidity or mortality between the two groups (Rabin et al. 2017). Both of these studies have all the shortcomings of being retrospective. Of note, a recent single-center, double-blind, randomized, controlled trial of albumin administration for a serum albumin level of less than 4.0 g/dL prior to OPCABG revealed a significant decrease in stage 1 acute kidney injury in the albumin group (Lee et al. 2016).

A well-designed, prospective, randomized trial would be required to address the question of whether albumin has a benefit on patient outcomes in cardiac surgery.

While this survey cannot elucidate the reasons behind why clinicians preferred a particular fluid type over another, the respondents did refer to “a more sustained volume response” as one of the major reasons for choosing a colloid for volume expansion.

It is noteworthy that more surgeons than anesthesiologists in this survey preferred HES solutions for patients needing volume expansion during CPB without bleeding (scenario 1). HES should be given with caution or possibly avoided altogether due to warnings from the FDA (United States Food and Drug Administration 2013), European Medicines Agency (2013), and Surviving Sepsis Campaign (Dellinger et al. 2013). In a recently published survey of fluid management in cardiac surgery that was conducted in 18 European countries, it was noted that the use of HES products has decreased dramatically in the last several years (Protsyk et al. 2017). The most commonly used fluids for intraoperative and postoperative fluid management were crystalloids, and if colloids were used, the colloids were used in combination with crystalloids. The colloids that were used most frequently in this recent European survey were the gelatins, followed by HES and albumin. The warnings about the risks of HES use in critically ill patients issued by the FDA and EMA may have had an effect on the use of HES in the USA as well. In the present survey, which was performed about 2 years after the regulatory agency warnings, HES was chosen as the fluid of first choice by less than 10% of the respondents in most of the clinical scenarios. In agreement with the European survey, crystalloids were the most commonly chosen first fluid choice in three of the five clinical scenarios. However, in the USA, gelatins are not available and the only non-HES colloid that is easily available is albumin.

### Limitations

A limitation of this study is that it describes current practices of fluid volume expansion in cardiovascular surgeries conducted only in the United States; these study results cannot be generalized to other countries in the rest of the world. We did not report on clinical outcomes, as this study is based on a survey of HCP preferences. We also recognize that the decision-making processes of HCPs are complex and may vary from the situations we put forth in the survey. While our study design cannot provide details on the underlying reasons for treatment decisions, the results allow HCPs to compare their own practice patterns to those of their colleagues, which could be helpful given the lack of expert consensus on fluid resuscitation in patients undergoing cardiovascular surgeries. The results in this survey also provide basic background information for the design of future trials that address complicated issues, such as fluid volume expansion strategies in the hypothetical scenarios we described.

## Conclusions

This study examined current practice patterns of fluid volume expansion in patients undergoing cardiac surgeries in the USA and found that fluid utilization varied depending on patient characteristics and clinical specialties of HCPs. Crystalloid fluid was most commonly chosen as the first-choice fluid for volume expansion. The most frequently used adjunct fluid to crystalloids was 5% albumin, which was also the most frequent first choice of IV fluid for patients needing volume expansion in the presence of blood loss during CPB when blood transfusion is not indicated (adequate Hb). In addition, perfusionists predominately preferred crystalloids to prime the CPB circuit; one third of the perfusionists preferred 25% albumin mixed with crystalloids for priming.

## Additional files

Additional file 1: Albumin Surgical Utilization Survey. (DOCX 415 kb)

Additional file 2: Figure S1. Frequency of adjunct fluid use for patients needing volume expansion during CPB when not experiencing significant blood loss when the first choice is crystalloids^a^ (scenario 1, *n* = 64). CPB, cardiopulmonary bypass; HES, hydroxyethyl starch. ^a^Responses to the following question: How often do you use each of the following as an adjunct to your first choice in a patient not experiencing significant blood loss when volume expansion is indicated during cardiovascular surgery with CPB? (JPEG 168 kb)

Additional file 3: Figure S2. Frequency of adjunct fluid use for patients needing volume expansion in the presence of blood loss during CPB when blood transfusion is not indicated (scenario 2) **a** when first fluid choice is 5% albumin^a^ (*n* = 52) and **b** when first fluid choice is crystalloids^b^ (*n* = 39). CPB cardiopulmonary bypass; Hb, hemoglobin; HES, hydroxyethyl starch. ^a^Responses to the following question: How often do you use each of the following as an adjunct to your first choice in a patient not experiencing significant blood loss when volume expansion is indicated during cardiovascular surgery with CPB? ^b^Responses to the following question: How often do you use each of the following as an adjunct to your first choice in a patient for volume expansion in the presence of blood loss when blood transfusion is not indicated (adequate Hb) during cardiovascular surgery with CPB? (ZIP 186 kb)

Additional file 4: Figure S3. Frequency of adjunct fluid use for patients needing volume maintenance during acute normovolemic hemodilution when first fluid choice is crystalloids^a^ (scenario 3, *n* = 78). HES, hydroxyethyl starch. ^a^Responses to the following question: How often do you use the following as an adjunct to your first choice for a patient for volume maintenance during acute normovolemic hemodilution (autologous blood collection)? (JPEG 167 kb)

Additional file 5: Figure S4. Frequency of adjunct fluid use for expansion during ECMO or VAD (scenario 4) **a** when first fluid choice is albumin 5%^a^ (*n* = 33^b^) and **b** when first fluid choice is crystalloids^a^ (*n* = 31^b^). ECMO, extracorporeal membrane oxygenation; HES, hydroxyethyl starch; VAD, ventricular assist device. ^a^Responses to the following question: How often do you use the following as an adjunct to your first choice for a patient who needs volume expansion during ECMO or VAD? ^b^No statistical tests were performed due to small sample size. (ZIP 169 kb)

Additional file 6: Figure S5. Frequency of adjunct fluid use for intraoperative volume expansion for OPCABG or TAVR when first fluid choice is crystalloids^a^ (scenario 5, *n* = 59). HES, hydroxyethyl starch; OPCAB, off-pump coronary artery bypass surgery; TAVR, transcatheter aortic valve replacement. ^a^Responses to the following question: How often do you use the following as an adjunct to your first choice for a patient who needs intraoperative volume expansion for OPCAB or TAVR? (JPEG 167 kb)

## Abbreviations

ANH: Acute normovolemic hemodilution
CABG: Coronary artery bypass graft surgery
COP: Colloid oncotic pressure
CPB: Cardiopulmonary bypass
ECMO: Extracorporeal membrane oxygenation
Hb: Hemoglobin
HCP: Health care professional
HES: Hydroxyethyl starches
ICU: Intensive care unit
IV: Intravenous
OPCABG: Off-pump coronary artery bypass graft surgery
TAVR: Transcatheter aortic valve replacement
VAD: Ventricular assist device
