# Supplementary material for: Fluid resuscitation practices in cardiac surgery patients in the USA: a survey of health care providers
Source: Perioper Med (Lond). 2017 Oct 19;6:15. doi: 10.1186/s13741-017-0071-6 (PMC5649061; doi:10.1186/s13741-017-0071-6)
Supplement: Supplementary file 1 — Albumin Surgical Utilization Survey. (DOCX 415 kb) [file 13741_2017_71_MOESM1_ESM.docx]

**Albumin Surgical Utilization Survey**

Nov. 2, 2015

V6

**Part 1: Demographics**

1. Which of the following is your primary specialty (select all that apply)?
   1. Cardiovascular Anesthesiology (adult)
   2. Cardiovascular Anesthesiology (pediatric)
   3. Cardiac Surgery
   4. Vascular Surgery
   5. Critical Care Medicine
   6. Perfusionist
   7. Other (Please specify _______________________________________________)
2. In which state or region is your primary practice located?

[Drop-down list of states]

[EXCLUDE / TERM PHYSICIANS IN Minnesota (MN), Vermont (VT), West Virginia (WVA), District of Columbia (DC) AND Massachusetts (MA)]

1. On average, what number of cardiac bypass surgeries do you perform or are involved in per month?

[RANGE; 0 – 100]

|_|_|_| cardiac bypass surgeries per month

1. What year did you finish your [IF CV ANESTHESIOLOGIST (Q1=A) OR SURGERY (Q1=C) INSERT “residency or fellowship” IF PERFUSIONIST (Q1=F) INSERT “perfusion”] training?

[Drop-down list of years] [RANGE 1951-2015]

[TERM IF 2013/2014/2015]

QUALIFYING CRITERIA: RESPONDENTS MUST MEET THE FOLLOWING SCREENING CRITERIA TO CONTINUE IN THE SURVEY:

1. Specialty must be one of the following: Target Quota (n)

- Cardiovascular Anesthesiology/Adult (Q1 = a) 50

- Surgery (Q1 = c) 50

- Perfusionist (Q1 = f) 50 .

**Total 150**

1. Practice must not be in one of the excluded states/areas listed in Q2
2. Must perform or be involved in at least 4 coronary bypass surgeries per month (Q3>=4).

D. Must have been in practice at least two years since residency (based on Q4)

|  |
| --- |

1. Which of the following best describes your primary practice setting?
   1. University Hospital
   2. Community Hospital
   3. VA Hospital
   4. Other (Specify)
2. Approximately how many beds in total does your primary hospital have?

[RANGE 0001 – 3000]

|_|_|_|_| beds in total

7. Which of the following best describes your hospital setting?

a. Urban

b. Suburban

c. Rural

**Part 2: Fluid and hemodynamic management**

1. This brief survey focuses on fluid and hemodynamic management in the OR and in the first 24-hour post-operative period. Please answer all questions as accurately as you can.

Using the scale below, please indicate how frequently you use each of the following fluids as **maintenance fluid** in the first 24-hour post-operative period, if at all.

|  | Always | Often | Sometimes | Rarely | Never |
| --- | --- | --- | --- | --- | --- |
| [RANDOMIZE ORDER] | 1 | 2 | 3 | 4 | 5 |
| NS (Normal saline) |  |  |  |  |  |
| DS (Dextrose saline) |  |  |  |  |  |
| LR (Lactated Ringer's solution) |  |  |  |  |  |
| Normosol/plasmalyte |  |  |  |  |  |
| Albumin |  |  |  |  |  |

1. Which of the following indicators (diagnostic tools) of volume status and the need for volume expansion do you use? (Please select all that apply.)
   1. Blood pressure
   2. Heart rate
   3. Cardiac output
   4. Central venous pressure
   5. Central venous saturation (ScvO2)
   6. Global end diastolic volume
   7. Mixed venous saturation (SvO2)
   8. Plethysmographic Waveform Variation
   9. Pulmonary capillary wedge pressure
   10. Pulse Pressure Variation or Systolic Pressure Variation
   11. Stroke Volume Variation
   12. Transesophageal echocardiography
   13. Noninvasive CO monitoring
   14. Urine output
   15. Electrolytes,
   16. Acid base status,
   17. Lactate
   18. Serum Albumin
   19. None. I depend solely on my clinical experience. [SKIP TO Q3]
   20. None of the above [SKIP TO Q3]

[ASK Q2B IF Q2/A through R]

2b. How frequently do you use each of the volume expansion indicators listed below?

|  | | Always | Often | Sometimes | Rarely | Never |
| --- | --- | --- | --- | --- | --- | --- |
| [DISPLAY ONLY INDICATORS SELECTED IN Q2.] [DISPLAY IN SAME ORDER AS IN Q2] | 1 | | 2 | 3 | 4 | 5 |
| Blood pressure |  | |  |  |  |  |
| Heart rate |  | |  |  |  |  |
| Cardiac output |  | |  |  |  |  |
| Central venous pressure |  | |  |  |  |  |
| Central venous saturation (ScvO2) |  | |  |  |  |  |
| Global end diastolic volume |  | |  |  |  |  |
| Mixed venous saturation (SvO2) |  | |  |  |  |  |
| Plethysmographic Waveform Variation |  | |  |  |  |  |
| Pulmonary capillary wedge pressure |  | |  |  |  |  |
| Pulse Pressure Variation or Systolic Pressure Variation |  | |  |  |  |  |
| Stroke Volume Variation |  | |  |  |  |  |
| Transesophageal echocardiography |  | |  |  |  |  |
| \| Urine output \|  \|  \|  \|  \|  \| \| --- \| --- \| --- \| --- \| --- \| --- \|   Electrolytes | | | | | | |
| Acid base status | | | | | | |
| Lactate | | | | | | |
| Serum Albumin | | | | | | |
|  | | | | | | |

1. Which of the following is your first choice for a patient who needs volume expansion during cardiovascular surgery with cardiopulmonary bypass (CPB) who is not experiencing significant blood loss?

[RANDOMIZE ORDER]

1. Albumin 5%

Keep/display together

Keep/display together

1. Albumin 25%
2. HES – Voluven
3. HES – hextend/hespan
4. Blood derived products other than albumin
5. Crystalloids
6. None; not applicable to my practice [ANCHOR; SKIP TO Q5]
7. How often do you use each of the following as an adjunct to your first choice in a patient not experiencing significant blood loss when volume expansion is indicated during cardiovascular surgery with cardiopulmonary bypass (CPB)?

| [DISPLAY ONLY CHOICES NOT SELECTED IN Q3.] | Always | Often | Sometimes | Rarely | Never |
| --- | --- | --- | --- | --- | --- |
|  | 1 | 2 | 3 | 4 | 5 |
| Albumin 5% |  |  |  |  |  |
| Albumin 25% |  |  |  |  |  |
| HES - Voluven |  |  |  |  |  |
| HES – hextend/hespan |  |  |  |  |  |
| Blood derived products other than albumin |  |  |  |  |  |
| Only crystalloids |  |  |  |  |  |

1. Which of the following is your first choice for a patient who needs volume expansion in the presence of blood loss when blood transfusion is not indicated (adequate Hb) during cardiovascular surgery with cardiopulmonary bypass (CPB)?

[RANDOMIZE ORDER]

1. Albumin 5%

Keep/display together

Keep/display together

1. Albumin 25%
2. HES – Voluven
3. HES – hextend/hespan
4. Blood derived products other than albumin
5. Crystalloids
6. None; not applicable to my practice [ANCHOR; SKIP TO Q7]
7. How often do you use each of the following as an adjunct to your first choice in a patient for volume expansion in the presence of blood loss when blood transfusion is not indicated (adequate Hb) during cardiovascular surgery with cardiopulmonary bypass (CPB)?

|  | Always | Often | Sometimes | Rarely | Never |
| --- | --- | --- | --- | --- | --- |
| [DISPLAY ONLY CHOICES NOT SELECTED IN Q5.] | 1 | 2 | 3 | 4 | 5 |
| Albumin 5% |  |  |  |  |  |
| Albumin 25% |  |  |  |  |  |
| HES - Voluven |  |  |  |  |  |
| HES – hextend/hespan |  |  |  |  |  |
| Blood derived products other than albumin |  |  |  |  |  |
| Only crystalloids |  |  |  |  |  |

1. Which of the following is your first choice for a patient for volume maintenance during acute normovolemic hemodilution (autologous blood collection)?

[RANDOMIZE ORDER]

1. Albumin 5%

Keep/display together

Keep/display together

1. Albumin 25%
2. HES – Voluven
3. HES – hextend/hespan
4. Blood derived products other than albumin
5. Crystalloids
6. None; not applicable to my practice [ANCHOR; SKIP TO Q9]
7. How often do you use the following as an adjunct to your first choice for a patient for volume maintenance during acute normovolemic hemodilution (autologous blood collection)?

| [DISPLAY ONLY CHOICES NOT SELECTED IN Q7.] | Always | Often | Sometimes | Rarely | Never |
| --- | --- | --- | --- | --- | --- |
|  | 1 | 2 | 3 | 4 | 5 |
| Albumin 5%  Keep/display together  Keep/display together |  |  |  |  |  |
| Albumin 25% |  |  |  |  |  |
| HES - Voluven |  |  |  |  |  |
| HES – hextend/hespan |  |  |  |  |  |
| Blood derived products other than albumin |  |  |  |  |  |
| Only crystalloids |  |  |  |  |  |

1. (Perfusionists (Q1=f)) Which of the following solutions is your first choice for priming the CPB circuit?

[RANDOMIZE ORDER]

1. Albumin 5% plus crystalloids

Keep/display together

Keep/display together

1. Albumin 25% plus crystalloids
2. HES – Voluven plus crystalloids
3. HES – hextend/hespan plus crystalloids
4. Blood derived products other than albumin, plus crystalloids
5. Crystalloids only

9b. (Perfusionists (Q1=f))

What is the total volume of solution you typically use to for priming the CPB circuit?

|_|_|_|_|cc total volume of priming solution

9c. [Perfusionists who say use blood derived products, HES, or crystalloids only (Q9=3,4,5 or 6 and Q1=f))]

Do you ever use albumin in priming?

1. Yes

2. No

9d. (IF EVER uses albumin in priming (Q9=1/2 OR Q9c=1)

In the box below, please describe how you use albumin when priming the CPB circuit, including albumin volume, whether you use albumin 5% or 25%, etc.

[LARGE TEXT BOX]

(All respondents)

1. Which of the following is your first choice for a patient who needs volume expansion during extracorporeal membrane oxygenation (ECMO) or ventricular assist device (VAD)?

[RANDOMIZE ORDER]

1. Albumin 5%

Keep/display together

Keep/display together

1. Albumin 25%
2. HES – Voluven
3. HES – hextend/hespan
4. Blood derived products other than albumin
5. Crystalloids
6. None; not applicable to my practice [ANCHOR; SKIP TO Q12]
7. [SHOW ONLY FOR PERFUSIONISTS] The physician makes this decision. [ANCHOR; SKIP TO Q12.]
8. How often do you use the following as an adjunct to your first choice for a patient who needs volume expansion during extracorporeal membrane oxygenation (ECMO) or ventricular assist device (VAD)?

|  | Always | Often | Sometimes | Rarely | Never |
| --- | --- | --- | --- | --- | --- |
|  | 1 | 2 | 3 | 4 | 5 |
| Albumin 5%  Keep/display together  Keep/display together |  |  |  |  |  |
| Albumin 25% |  |  |  |  |  |
| HES - Voluven |  |  |  |  |  |
| HES – hextend/hespan |  |  |  |  |  |
| Blood derived products other than albumin |  |  |  |  |  |
| Only crystalloids |  |  |  |  |  |

1. Which of the following is your first choice for a patient who needs intraop volume expansion for off-pump CABG (OPCAB) or transcatheter aortic valve replacement (TAVR)?

[RANDOMIZE ORDER]

1. Albumin 5%

Keep/display together

Keep/display together

1. Albumin 25%
2. HES – Voluven
3. HES – hextend/hespan
4. Blood derived products other than albumin
5. Crystalloids
6. None; not applicable to my practice [ANCHOR; SKIP TO Q14]
7. [SHOW ONLY FOR PERFUSIONISTS] The physician makes this decision. [ANCHOR; SKIP TO Q14.]
8. How often do you use the following as an adjunct to your first choice for a patient who needs intraop volume expansion for off-pump CABG (OPCAB) or transcatheter aortic valve replacement (TAVR)?

|  | Always | Often | Sometimes | Rarely | Never |
| --- | --- | --- | --- | --- | --- |
|  | 1 | 2 | 3 | 4 | 5 |
| Albumin 5% |  |  |  |  |  |
| Albumin 25% |  |  |  |  |  |
| HES - Voluven |  |  |  |  |  |
| HES – hextend/hespan |  |  |  |  |  |
| Blood derived products other than albumin |  |  |  |  |  |
| Only crystalloids |  |  |  |  |  |

1. Using the scale below, please indicate how important each of the following is in terms of your reasons for using colloids for volume expansion.

|  | Not Important | Somewhat Important | Important | Very Important | Absolutely Essential |
| --- | --- | --- | --- | --- | --- |
| [RANDOMIZE ORDER] | 1 | 2 | 3 | 4 | 5 |
| More sustained volume expansion with colloids |  |  |  |  |  |
| Faster volume expansion with colloids |  |  |  |  |  |
| Less interstitial edema with colloids |  |  |  |  |  |
| Less weight gain with colloids |  |  |  |  |  |
| Better respiratory function with colloids |  |  |  |  |  |

[ASK Q15 IF TWO OR MORE PROPERTIES IN Q14 ARE RATED EQUALLY AND RATED MOST IMPORANT RELATIVE TO OTHER PROPERTIES. ERROR MESSAGE: “Please revise your responses on this page and select only one item per rank order.”

1. Please rank order the following in terms of their importance to your reasons for using colloids for volume expansion, with 1 indicating the “Most important” reason.

SHOW ONLY NUMBER OF RANK POSITIONS EQUAL TO NUMBER OF PROPERTIES SHOWN

| [SHOW ONLY PROPERTIES FROM Q14 RATED  Most Important  2  1  EQUALLY AND RATED MORE IMPORTANT  RELATIVE TO OTHER PROPERTIES] | 3 | 4 | 5 |
| --- | --- | --- | --- |
| [DISPLAY IN SAME ORDER AS IN Q14] |  |  |  |
| More sustained volume expansion with colloids |  |  |  |
| Faster volume expansion with colloids |  |  |  |
| Less interstitial edema with colloids |  |  |  |
| Less weight gain with colloids |  |  |  |
| Better respiratory function with colloids |  |  |  |

1. Which of the following do you most often use to bolus for volume expansion?

[ROTATE ORDER]

a. Colloid

b. Crystalloid

17a. When indicated, what volume of colloid bolus do you typically use for volume expansion as your standard dose?

[RANGE 001 – 9999]

|_|_|_|_| ml

17b. When indicated, what volume of crystalloid bolus do you typically give for volume expansion as your standard dose?

[RANGE 001 – 9999]

|_|_|_|_| ml

1. How common is it in your practice to see each of following adverse reactions to albumin?

| [RANDOMIZE ORDER] | Common  (1 in 10) | Rare  (1 in 100) | | Very Rare  (1 in 1000+) | | Have never seen it | |  |
| --- | --- | --- | --- | --- | --- | --- | --- | --- |
| Rash |  | |  | |  | |  | |
| Hypotension |  | |  | |  | |  | |
| Anaphylaxis |  | |  | |  | |  | |
| Pulmonary edema |  | |  | |  | |  | |

1. Using the scale below, please indicate how important each of the following non-oncotic properties of albumin is to you in treating your patients.

| Not  Important | | | Somewhat Important | | Important | | Very  Important | | Absolutely Essential | | I am not aware of this property | |
| --- | --- | --- | --- | --- | --- | --- | --- | --- | --- | --- | --- | --- |
| [RANDOMIZE ORDER] 1 | | | 2 | | 3 | | 4 | | 5 | | 99 | |
| Transport of metabolites | | |  | |  | |  | |  | |  | |
| Free radical scavenging | | |  | |  | |  | |  | |  | |
| Anti-inflammatory effects | | |  | |  | |  | |  | |  | |
| Maintenance of vascular integrity | | |  | |  | |  | |  | |  | |
| Antithrombotic effects | | |  | |  | |  | |  | |  | |
| Drug binding |  |  | |  | |  | |  | |  | |  |

[ASK Q20 IF TWO OR MORE PROPERTIES IN Q19 ARE RATED EQUALLY AND RATED MORE IMPORANT RELATIVE TO OTHER PROPERTIES.] DO NOT INCLUDE ANY PROPERTY RANKED 99. ERROR MESSAGE: “Please revise your responses on this page and select only one item per rank order.”

1. Please rank the following non-oncotic properties of albumin in order of their importance to you in treating your patients, with 1 indicating the “Most important” reason.

SHOW ONLY NUMBER OF RANK POSITIONS EQUAL TO NUMBER OF PROPERTIES SHOWN

|  | | | Most  Important | |  |  | |  | | |  |  | |  |
| --- | --- | --- | --- | --- | --- | --- | --- | --- | --- | --- | --- | --- | --- | --- |
| [SHOW ONLY PROPERTIES FROM Q19 RATED EQUALLY AND RATED MORE IMPORTANT RELATIVE TO OTHER PROPERTIES] | | | 1 | | 2 | 3 | | 4 | | | 5 | 6 | |  |
| Transport of metabolites | | |  | |  |  | |  | | |  |  | |  |
| Free radical scavenging | | |  | |  |  | |  | | |  |  | |  |
| Anti-inflammatory effects | | |  | |  |  | |  | | |  |  | |  |
| Maintenance of vascular integrity | | |  | |  |  | |  | | |  |  | |  |
| Antithrombotic effects | | |  | |  |  | |  | | |  |  | |  |
| Drug binding |  |  | |  | | |  | |  |  | | |  | |

20b. Which of the following best describes the level of influence you have, in general, on decisions to use albumin?

1. Not at all influential
2. Somewhat influential
3. Influential
4. Very influential
5. Extremely influential

Questions 21A – 21E are only asked to respondents who rarely or never use albumin 5%. All others skip to Q22.

[ONLY ASK 21A IF Q3 NE 1 AND Q4_1=4 OR 5 (SELECTED “RARELY” OR “NEVER” FOR “ALBUMIN 5%” IN Q4)]

21A. You indicated that you rarely or never use albumin 5% for a patient who needs volume expansion during cardiovascular surgery with cardiopulmonary bypass (CPB) who is not experiencing significant blood loss?

Please briefly tell us why you rarely or never use albumin 5% for this type of patient.

[Large text box]

[ONLY ASK 21B IF Q5 NE 1 AND Q6_1=4 OR 5 (SELECTED “RARELY” OR “NEVER” FOR “ALBUMIN 5%” IN Q6)]

21B. You indicated that you rarely or never use albumin 5% for a patient for volume expansion in the presence of blood loss when blood transfusion is not indicated (adequate Hb) during cardiovascular surgery with cardiopulmonary bypass (CPB).

Please briefly tell us why you rarely or never use albumin 5% for this type of patient.

[Large text box]

[ONLY ASK 21C IF Q7 NE 1 AND Q8_1=4 OR 5 (SELECTED “RARELY” OR “NEVER” FOR “ALBUMIN 5%” IN Q8)]

21C. You indicated that you rarely or never use albumin 5% for a patient for volume maintenance during acute normovolemic hemodilution (autologous blood collection).

Please briefly tell us why you rarely or never use albumin 5% for this type of patient.

[Large text box]

[ONLY ASK 21D IF Q10 NE 1 AND Q11_1=4 OR 5 (SELECTED “RARELY” OR “NEVER” FOR “ALBUMIN 5%” IN Q11)]

21D. You indicated that you rarely or never use albumin 5% for a patient who needs volume expansion during extracorporeal membrane oxygenation (ECMO) or ventricular assist device (VAD).

Please briefly tell us why you rarely or never use albumin 5% for this type of patient.

[Large text box]

[ONLY ASK 21E IF Q12 NE 1 AND Q13_1=4 OR 5 (SELECTED “RARELY” OR “NEVER” FOR “ALBUMIN 5%” IN Q13)]

21E. You indicated that you rarely or never use albumin 5% for a patient who needs intraop volume expansion for off-pump CABG (OPCAB) or transcatheter aortic valve replacement (TAVR).

Please briefly tell us why you rarely or never use albumin 5% for this type of patient.

[Large text box]

1. Thank you for your interest and time in completing this survey. [END]
