# Supplementary figures and images for: Fluid resuscitation practices in cardiac surgery patients in the USA: a survey of health care providers
Source: Perioper Med (Lond). 2017 Oct 19;6:15. doi: 10.1186/s13741-017-0071-6 (PMC5649061; doi:10.1186/s13741-017-0071-6)

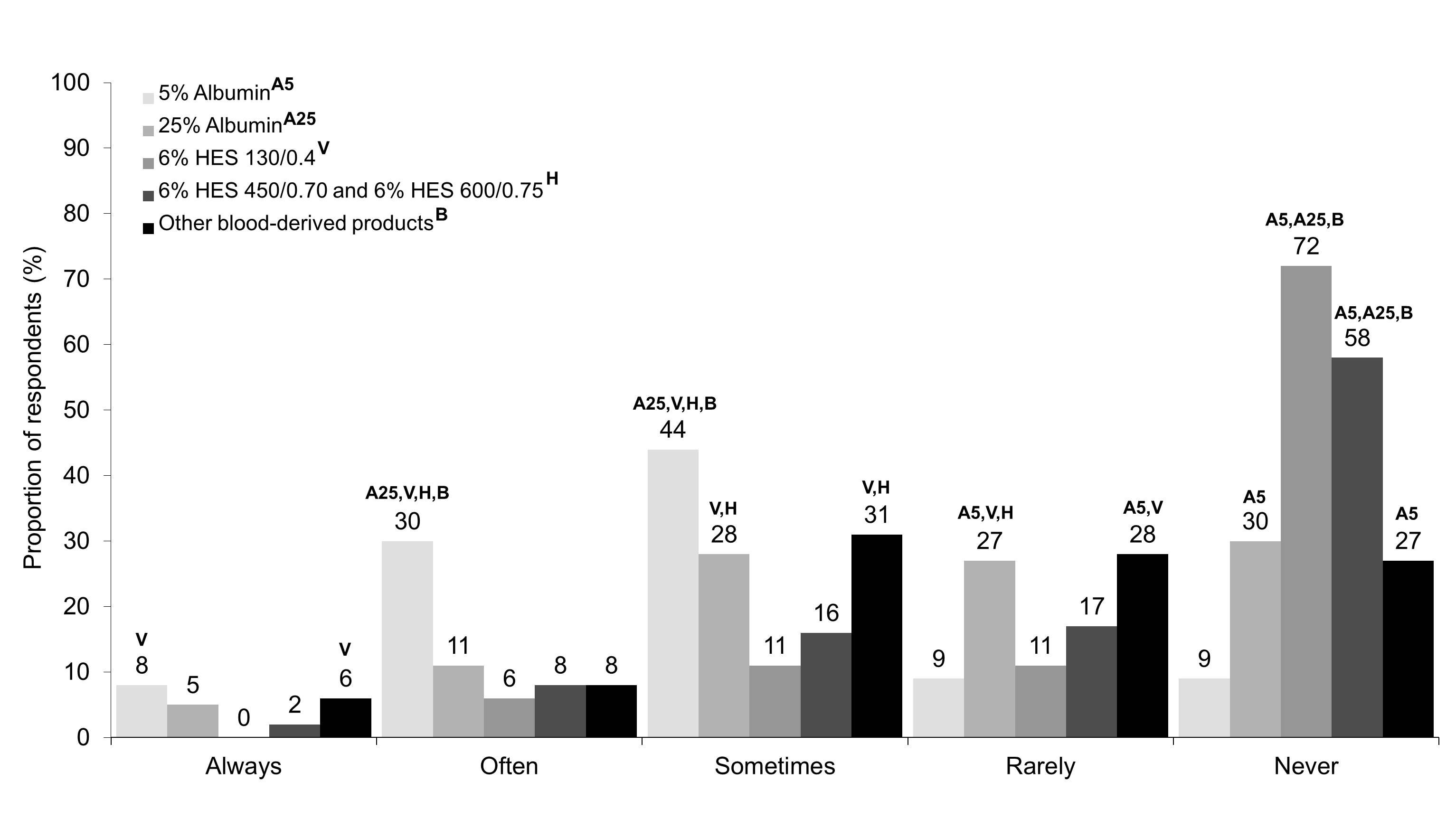

Supplement: Supplementary file 2 — Frequency of adjunct fluid use for patients needing volume expansion during CPB when not experiencing significant blood loss when the first choice is crystalloidsa (scenario 1, n = 64). CPB, cardiopulmonary bypass; HES, hydroxyethyl starch. aResponses to the following question: How often do you use each of the following as an adjunct to your first choice in a patient not experiencing significant blood loss when volume expansion is indicated during cardiovascular surgery with CPB? (JPEG 168 kb) [file 13741_2017_71_MOESM2_ESM.jpg]

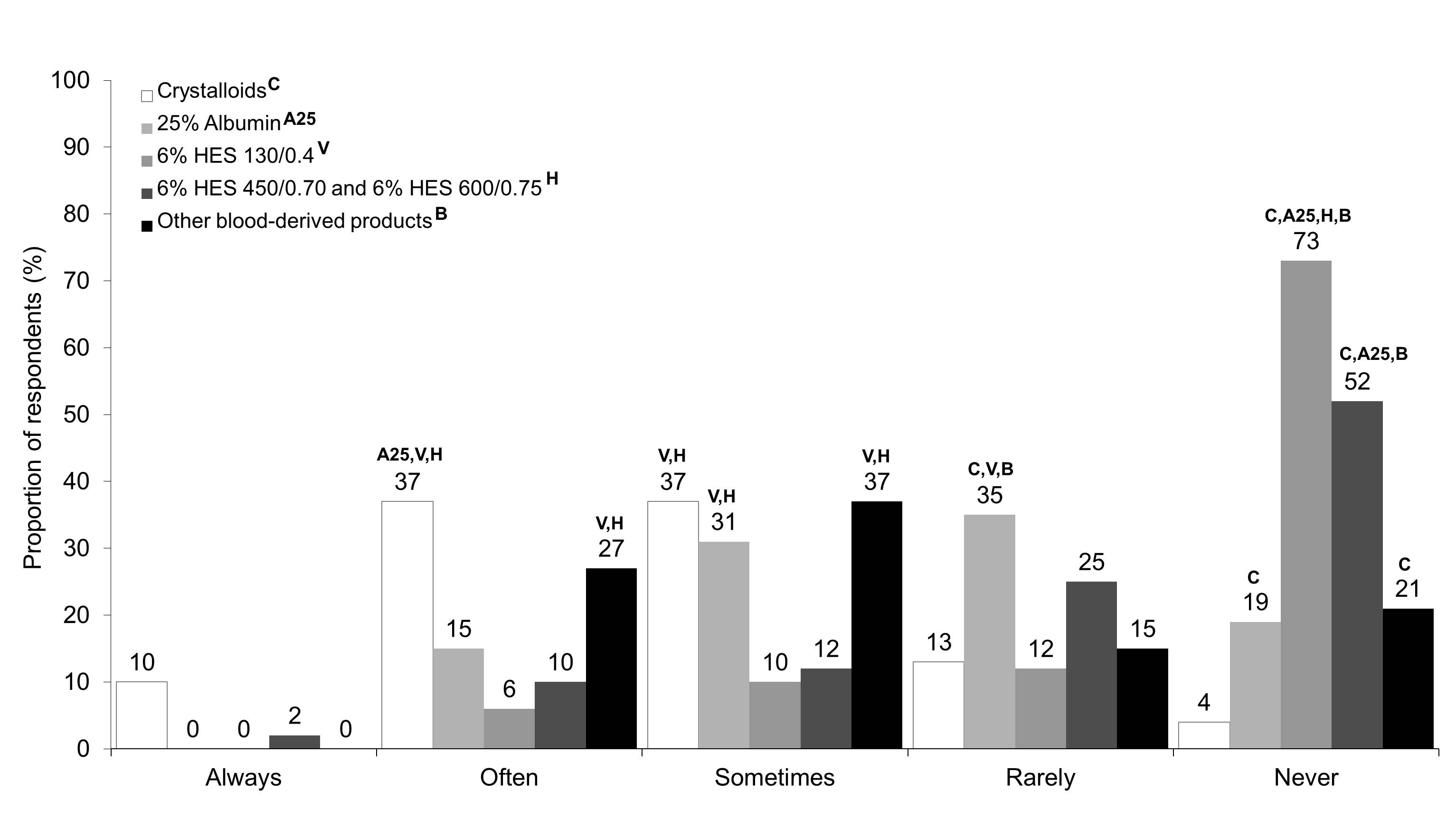

Supplement: Supplementary file 3 — Frequency of adjunct fluid use for patients needing volume expansion in the presence of blood loss during CPB when blood transfusion is not indicated (scenario 2) a when first fluid choice is 5% albumina (n = 52) and b when first fluid choice is crystalloidsb (n = 39). CPB cardiopulmonary bypass; Hb, hemoglobin; HES, hydroxyethyl starch. aResponses to the following question: How often do you use each of the following as an adjunct to your first choice in a patient not experiencing significant blood loss when volume expansion is indicated during cardiovascular surgery with CPB? bResponses to the following question: How often do you use each of the following as an adjunct to your first choice in a patient for volume expansion in the presence of blood loss when blood transfusion is not indicated (adequate Hb) during cardiovascular surgery with CPB? (ZIP 186 kb) [file 13741_2017_71_MOESM3_ESM.zip › Additional file 2/CVSxSurvey_SupplFig2A.JPG]

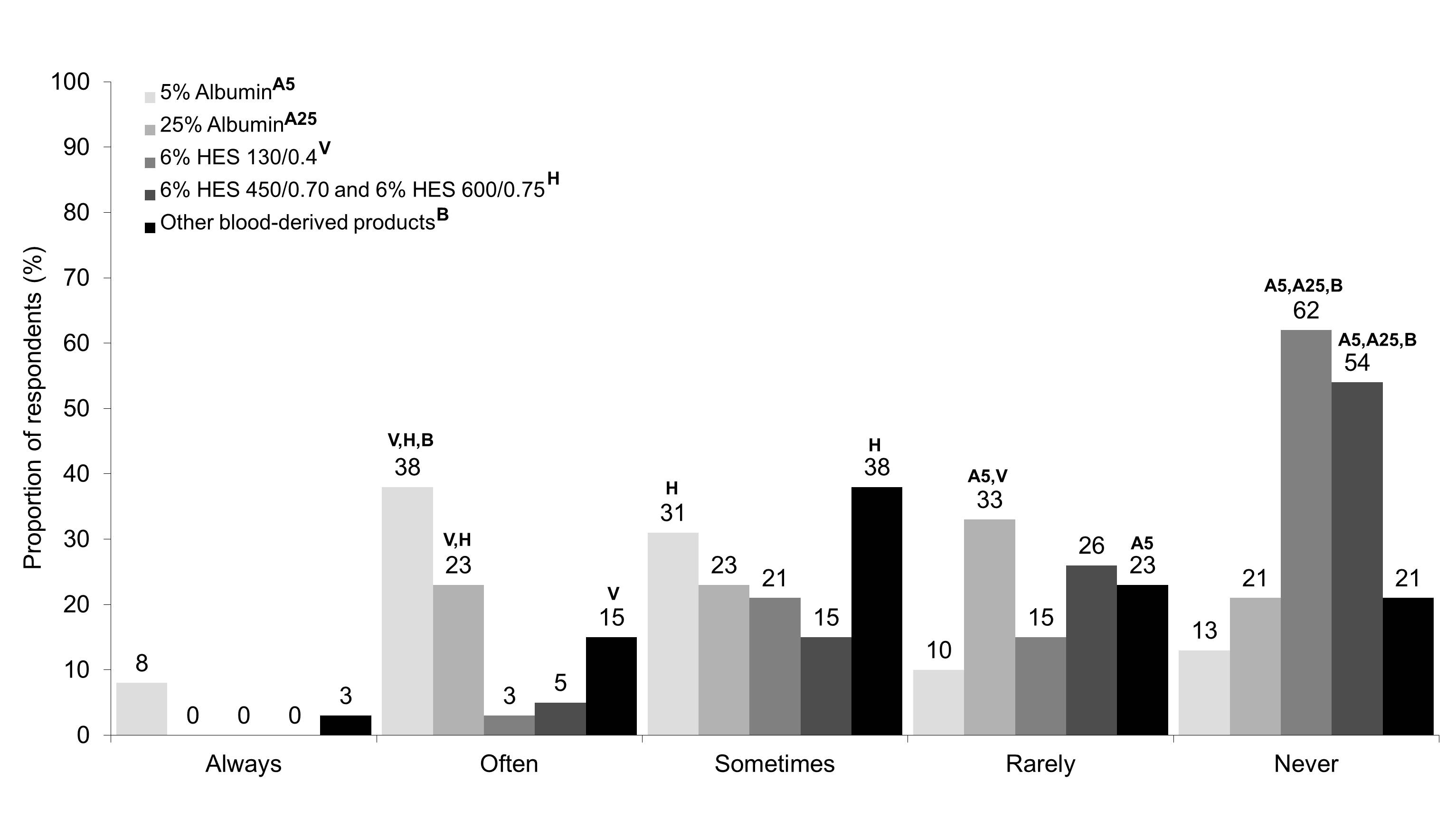

Supplement: Supplementary file 3 — Frequency of adjunct fluid use for patients needing volume expansion in the presence of blood loss during CPB when blood transfusion is not indicated (scenario 2) a when first fluid choice is 5% albumina (n = 52) and b when first fluid choice is crystalloidsb (n = 39). CPB cardiopulmonary bypass; Hb, hemoglobin; HES, hydroxyethyl starch. aResponses to the following question: How often do you use each of the following as an adjunct to your first choice in a patient not experiencing significant blood loss when volume expansion is indicated during cardiovascular surgery with CPB? bResponses to the following question: How often do you use each of the following as an adjunct to your first choice in a patient for volume expansion in the presence of blood loss when blood transfusion is not indicated (adequate Hb) during cardiovascular surgery with CPB? (ZIP 186 kb) [file 13741_2017_71_MOESM3_ESM.zip › Additional file 2/CVSxSurvey_SupplFig2B.JPG]

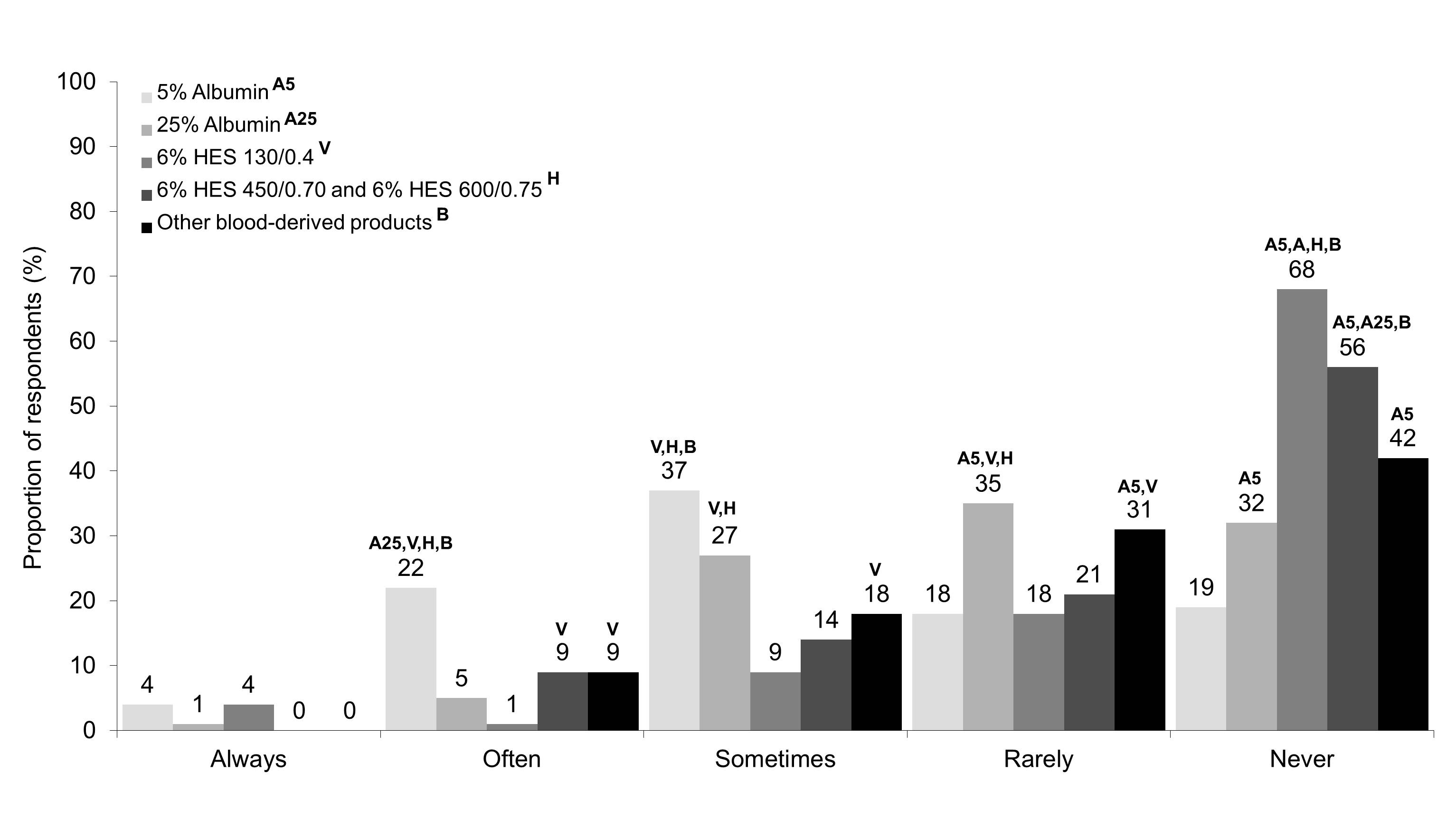

Supplement: Supplementary file 4 — Frequency of adjunct fluid use for patients needing volume maintenance during acute normovolemic hemodilution when first fluid choice is crystalloidsa (scenario 3, n = 78). HES, hydroxyethyl starch. aResponses to the following question: How often do you use the following as an adjunct to your first choice for a patient for volume maintenance during acute normovolemic hemodilution (autologous blood collection)? (JPEG 167 kb) [file 13741_2017_71_MOESM4_ESM.jpg]

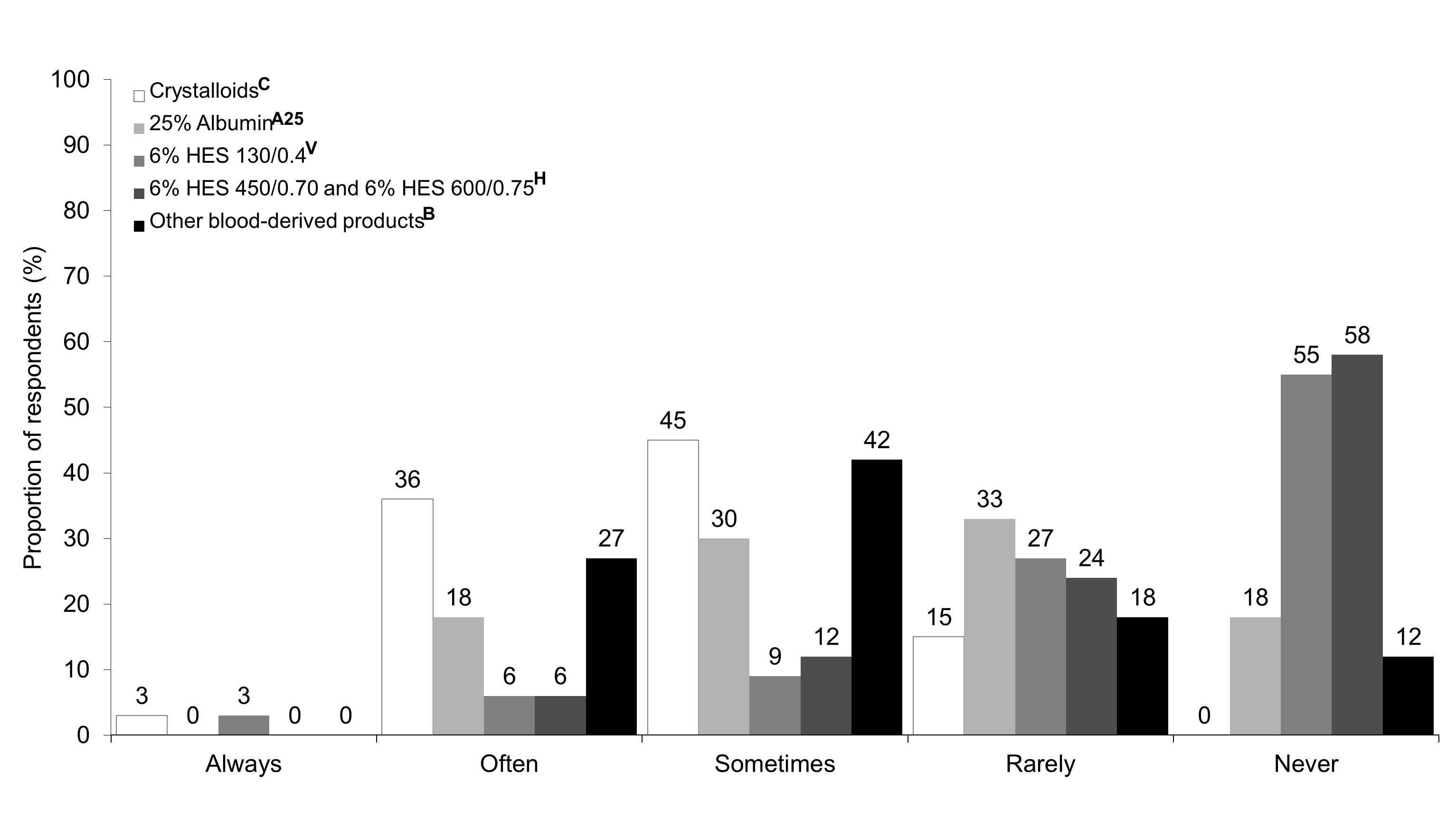

Supplement: Supplementary file 5 — Frequency of adjunct fluid use for expansion during ECMO or VAD (scenario 4) a when first fluid choice is albumin 5%a (n = 33b) and b when first fluid choice is crystalloidsa (n = 31b). ECMO, extracorporeal membrane oxygenation; HES, hydroxyethyl starch; VAD, ventricular assist device. aResponses to the following question: How often do you use the following as an adjunct to your first choice for a patient who needs volume expansion during ECMO or VAD? bNo statistical tests were performed due to small sample size. (ZIP 169 kb) [file 13741_2017_71_MOESM5_ESM.zip › Additional file 4/CVSxSurvey_SupplFig4A.JPG]

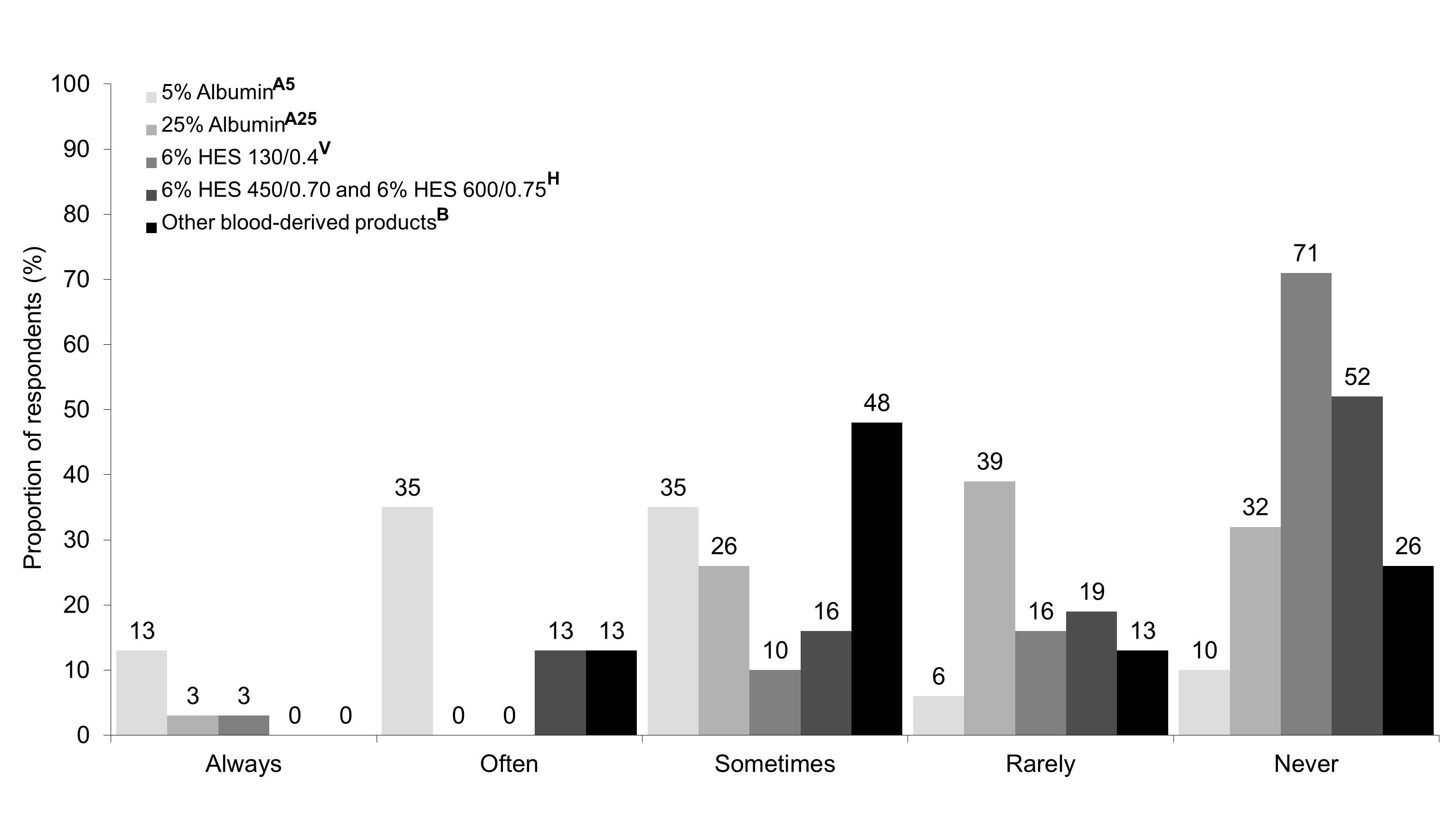

Supplement: Supplementary file 5 — Frequency of adjunct fluid use for expansion during ECMO or VAD (scenario 4) a when first fluid choice is albumin 5%a (n = 33b) and b when first fluid choice is crystalloidsa (n = 31b). ECMO, extracorporeal membrane oxygenation; HES, hydroxyethyl starch; VAD, ventricular assist device. aResponses to the following question: How often do you use the following as an adjunct to your first choice for a patient who needs volume expansion during ECMO or VAD? bNo statistical tests were performed due to small sample size. (ZIP 169 kb) [file 13741_2017_71_MOESM5_ESM.zip › Additional file 4/CVSxSurvey_SupplFig4B.JPG]

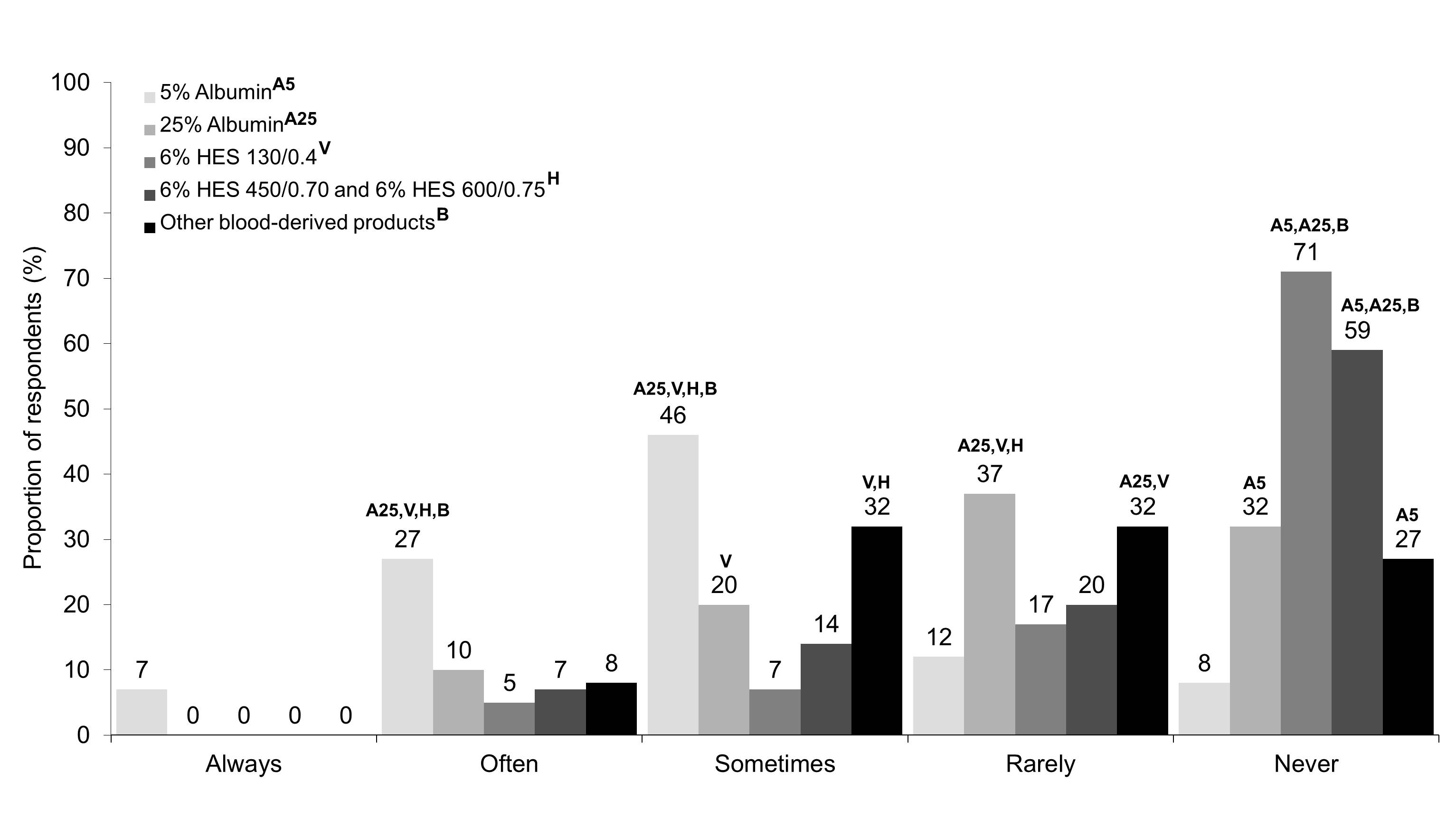

Supplement: Supplementary file 6 — Frequency of adjunct fluid use for intraoperative volume expansion for OPCABG or TAVR when first fluid choice is crystalloidsa (scenario 5, n = 59). HES, hydroxyethyl starch; OPCAB, off-pump coronary artery bypass surgery; TAVR, transcatheter aortic valve replacement. aResponses to the following question: How often do you use the following as an adjunct to your first choice for a patient who needs intraoperative volume expansion for OPCAB or TAVR? (JPEG 167 kb) [file 13741_2017_71_MOESM6_ESM.jpg]
